# Short-Term Resistance Training Supported by Whole-Body Cryostimulation Induced a Decrease in Myostatin Concentration and an Increase in Isokinetic Muscle Strength

**DOI:** 10.3390/ijerph17155496

**Published:** 2020-07-30

**Authors:** Joanna Jaworska, Ewa Rodziewicz-Flis, Jakub Kortas, Marta Kozłowska, Katarzyna Micielska, Anna Babińska, Radosław Laskowski, Giovanni Lombardi, Ewa Ziemann

**Affiliations:** 1 Department of Physiology, Gdansk University of Physical Education and Sport, Kazimierza Gorskiego 1, 80-336 Gdansk, Poland; joanna.jaworska@awf.gda.pl (J.J.); kozl.marta@gmail.com (M.K.); radoslaw.laskowski@awf.gda.pl (R.L.); 2 Department of Physical Therapy and Biological Regeneration, Gdansk University of Physical Education and Sport, Kazimierza Gorskiego 1, 80-336 Gdansk, Poland; ewa.rodziewicz@awf.gda.pl; 3 Department of Sport, Gdansk University of Physical Education and Sport, Kazimierza Gorskiego 1, 80-336 Gdansk, Poland; jakub.kortas@awf.gda.pl; 4 Department of Physical Education and Lifelong sports, Poznan University of Physical Education, Krolowej Jadwigi 27/39, 61-871 Poznan, Poland; micielska@awf.poznan.pl; 5 Department of Endocrinology and Internal Medicine, Medical University, Marii Sklodowskiej-Curie 3, 80-001 Gdansk, Poland; a.mail@wp.pl; 6 Department of Athletics, Strength and Conditioning, Poznan University of Physical Education, Krolowej Jadwigi 27/39, 61-871 Poznan, Poland; giovanni.lombardi@grupposandonato.it; 7 IRCCS Istituto Ortopedico Galeazzi, Lab Experimental Biochemistry & Molecular Biology, Via Riccardo Galeazzi, 4, 20161 Milano, Italy

**Keywords:** adaptation, growth factors, myokines, irisin, recovery

## Abstract

The study aimed to determine whether combining cryostimulation with resistance training would effectively increase muscle strength, and if so, whether this adaptation would be related to changes in circulating levels of exerkines (i.e., mediators of systemic adaptation to exercise). Twenty-five students completed 12 sessions of resistance training, each followed by either cryostimulation (*n* = 15, 3 min exposure at −110 °C) or passive recovery (*n* = 10). Prior to and post this intervention, participants performed two eccentric cycling bouts (before and after training). At these points, serum concentrations of muscle damage marker (myoglobin), exerkines (interleukin 6 (IL-6), interleukin 15 (IL-15), irisin, brain-derived neurotrophic factor), hypertrophy-related factors (myostatin, insulin-like growth factor 1), and muscle strength were measured. The applied procedure reduced the physiological burden of the second eccentric cycling bout and myoglobin concentrations only in the group subject to cryostimulation. The same group also exhibited decreased levels of myostatin (from 4.7 ± 1.7 to 3.8 ± 1.8 ng·mL^−1^, *p* < 0.05). A significant and large interaction between the group × time was noted in IL-15 concentration (*p* = 0.01, $\eta_{p}^{2}=0.27$). Training and cryostimulation induced a positive and likely significant improvement of isokinetic muscle strength. Altogether, obtained results support the claim that resistance training combined with cold exposure modified muscle strength through modulation of myostatin and IL-15 concentrations.

## 1. Introduction

Resistance training is a form of physical activity particularly popular among exercise novices. Compared to running or cycling, however, it is more technically complex and risky for individuals unaccustomed to exercise. A lack of experience, limited motor control, or incorrect exercise technique runs the risk of injury when left unsupervised. At the same time, individuals may experience exercise-induced muscle damage (EIMD) [1], which can hinder exercising for several days, possibly discouraging beginners to maintain physical activity. 

However, EIMD is an integral part of the high-intensity and resistance training process [2], which may be associated with delayed-onset muscle soreness (DOMS), muscle weakness, and a decreased range of motion. The terms EIMD and DOMS are often, although erroneously, used interchangeably. Indeed, EIMD can be, although not necessarily, the cause of DOMS when the intensity of exercise exceeds the level to which the subject is accustomed. On the other hand, muscle damage induced by low- and moderate-intensity exercise triggers the regenerative response that is not associated with soreness [3]. Ultimately, EIMD can affect physical performance with its effects lasting even for several days [4]; however, many studies showed that excessive EIMD, associated with eccentric work (also called negative muscle actions) [5] in resistance training protocols, did not promote additional gains in muscle strength [6]. EIMD is associated with high levels of circulating myoglobin, creatine kinase, and interleukin 6 (IL-6) [4]. This pro-inflammatory state in skeletal muscles stimulates expression and signaling of cytokines and growth factors, which, in turn, may trigger proliferation of satellite cells [7]. 

Among several interventions (including heat, manual therapy, and compression garments), cold treatment is a popular strategy to reduce EIMD symptoms [8]. Easily accessible, cold therapies such as ice bags, cold water immersion (CWI) [9], winter swimming [10], partial- and whole-body cryostimulation (CRY) [11,12] are particularly popular among professional sportsmen and late sport enthusiasts [13,14,15]. Nevertheless, recent reports have put forward a concept that resistance training combined with cold treatment may impair muscle adaptation to exercise. For instance, CWI (20 min at 10 °C) applied immediately after each session of moderate-intensity resistance training resulted in significantly lower gains in muscle strength and muscle thickness compared to passive rest [16]. The applied protocol lasted 6 weeks and consisted of 18 training sessions with a workload of about 70–80% of single-repetition maximum (1-RM) [16]. Furthermore, Roberts et al. reported attenuated muscle adaptation to long-term resistance training (12 weeks; 24 sessions; workload 8, 10, and 12 RM) [17], when training and CWI were combined (10 min at 10 °C). Nevertheless, data on how to use different cold procedures interchangeably are inconsistent. Costello and co-workers compared effects of a single CWI (4 min at 8 °C) and CRY session (3.40 min at −110 °C), and found that their impacts on muscle and core temperatures were similar, differing only in the aspect of skin cooling [18]. 

Recent reports indicated that cold therapies are not only effective in reducing inflammation and muscle pain after EIMD [19] but also modulate expression and release of a plethora of circulating growth factors [20]. What is more, cold exposure may change concentrations of amino acids, proteins released from muscle mass-myokines [20,21], and other proteins stimulated by exercise called “exerkines”, which are considered as the main mediators of systemic adaptation to exercise [22]. Broatch et al. reported neither positive nor negative effects of CWI combined with resistance training on the adaptation process, strongly emphasizing the importance of investigating different protocols of cold therapy during real-world resistance training [23]. The range of changes in growth factors and muscle functions in response to resistance training combined with cold treatment in beginners remains an open question.

In light of this background, the aim of this study was to investigate whether combining resistance training with cold treatment applied one day post the training session, would have an additional beneficial effect on muscle strength, muscle damage, and blood exerkine profile (such as interleukins (IL-6, IL-15), brain-derived neurotrophic factor (BDNF), insulin-like growth factor 1 (IGF-1), myostatin, irisin) as they are the main mediators of systemic adaptation to exercise [24].

## 2. Materials and Methods 

**Figure 1:**
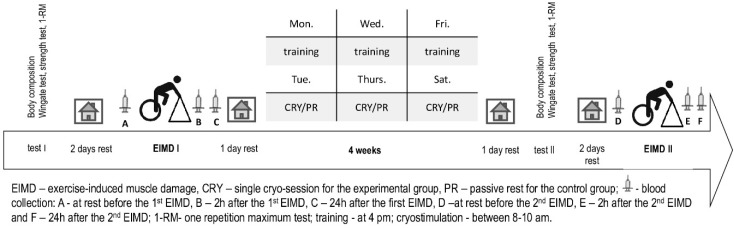
The schedule of the experiment.

In our study, we assessed the effects of different recovery strategies during one month of strength training program among young, healthy participants, who had never undergone strength training. In the experimental group, we used whole-body cryostimulation (CRY), a day after each training unit, while the control group (CON) recovered passively. Muscle strength, adaptation to EIMD (based on the eccentric muscle actions), and blood exerkines concentration were tested at baseline and after completion of the training program. We considered the necessary number of training units in order to induce adaptive changes [6]. The study design is provided in Figure 1.

### 2.1. Subjects

A total of 30 healthy (17 women and 13 men), untrained students, from the University of Physical Education, volunteered to take part in the investigation (age 20.3 ± 1.1, body weight 73.7 ± 11.7 kg). Twenty-five participants completed the whole intervention (5 students did not finish the experiment due to personal reasons). Prior to commencing the experiment, the subjects were informed about the procedures, risks, and benefits of the study. Eligible subjects were asked to give their written consent to participate and underwent a medical examination. After performing the first EIMD, participants were randomly assigned to one of the two groups: the experimental group, that was treated with whole-body cryostimulation (CRY; *n* = 15, body mass index 23.7 ± 3.2 kg·m^−2^) and the passively recovering control group (CON; *n* = 10, body mass index 23.2 ± 2 kg·m^−2^). None of the participants had previous experience with CRY in the year preceding the study. The participants were asked to eat in the same place (open-access canteen in the dorm, where they were living) as well as not to take any supplementation during the study. Additionally, they were instructed by the professional dietitian to eat 1.2 g·kg^−1^ of protein per day. During the study, none of the subjects was involved in any organized or recreational physical activity, other than the protocol. 

### 2.2. Ethics Statement 

This study was approved by the Bioethical Committee of the Regional Medical Society in Gdansk KB-28/17. Before the study initiation, the participants received a verbal description of the experiment, and informed consent was obtained from all participants. 

### 2.3. Test-Day Design

The subjects had to successively participate in the following tests, three days prior to commencing the experiment: anthropometric measurements, a muscle strength test, a 1-RM test (leg extension test, hamstring curl, and leg press). Additionally, in order to establish the individual intensity of eccentric cycling exercise on Cyclus2, the maximal anaerobic power (Wingate Anaerobic Test (WAnT)) was determined. WAnT was performed on a mechanical cycle ergometer (Monark Ergomedic 884E Sprint Bike, Sweden) The protocol started with the standardized 5 min warm-up (1.0 W·kg^−1^ of body mass) including two all-out sprints (of approximately 5 s each). After the warm-up, the participants took 2 min of rest before performing the 30 s all-out supramaximal concentric work. The applied external load equaled 7.5% of the individual body mass. The subjects were instructed to pedal at their maximum rate throughout the test [25]. After two days of rest, our participants performed the single bout of eccentric work on a cycling ergometer (negative work) [5]. Then, all of them participated in training program and underwent the specific recovery method. The day after the last training session, the participants repeated the mentioned measurements (Figure 1).

### 2.4. Anthropometric Measurements

Body mass and composition were estimated using a multi-frequency impedance plethysmography body composition analyzer (In Body 720, Biospace, Korea). Body mass was measured after an overnight fast, 12 h after the last meal and drink [26]. 

### 2.5. Muscle Strength Assessment

Isometric and isokinetic knee muscle functions were measured using the Biodex System 4 dynamometer (Biodex Medical System, Inc. Shirley, NY, USA). All tests were conducted in the sitting position (with the hip joint at an angle of 90°) with the arms folded across the chest, or the hands clasped in front of the body, and the trunk and lower limbs stabilized with a belt. The participants were asked to hold their tongue extended up to the palatine spot during the execution of all tests [27]. The subjects received standardized verbal instructions before the test and verbal encouragement throughout. Measurements of the peak torque were obtained for the extension of the knee joints (of both legs, separately) in conditions of a 5 s isometric contraction. The subjects were asked to contract “as hard as possible” (to the maximum) to obtain their maximal peak torque. Each peak torque measurement was obtained 3 times with a 1 min break between them. The extension of the knee was measured during isometric contractions at 90° of knee flexion. After completing the isometric strength test on both legs, the participants immediately proceeded to the isokinetic strength testing of the knee extensors. This test was performed at a velocity of 90°·s^−1^ (Nm) and repeated 3 times. Measurements of the peak torque and average power were analyzed in isokinetic conditions

### 2.6. Muscle Adaptation Assessment

In order to assess muscle adaptation to resistance training and evaluate the tolerance to EIMD, both groups completed the two sessions on an eccentric cycle ergometer (Cyclus2 Eccentric Trainer, Germany) (Figure 1). Two months before the main experiment, we had established the exercise protocol (intensity and duration) to induce muscle damage. To avoid muscle adaptation to eccentric contraction, a different group of volunteers (with similar anthropometric features and physical abilities and in the same age range) participated in the eccentric cycling trail sessions. They also received the information about the experiment and they signed an informed consent. Based on subjective feedback of feeling pain, values of serum creatine kinase activity and myoglobin concentrations (and mutual colorations between them), 50% of maximal anaerobic power was chosen for the main experimental session as workload. 

In this study, eccentric cycling began with 1 min of initiation/induction at a workload of 1.5 W·kg^−1^, followed by 10 min of exercise with a pedaling cadence ranging between 50 and 65 rev·min^−1^, at an individual workload of 50% of the values of maximal anaerobic power output. To assess the physiological cost of exercise, the heart rate (HR) was monitored throughout the exercise (Garmin, Olathe, KS, USA). Muscle pain in the lower limbs was assessed using the visual analog scale (VAS), in which 0 represents the feeling of no pain and 10 represents the feeling of maximal pain. The participants gave their subjective rating of muscle soreness immediately, 2 h, and 24 h after performing the bouts of EIMD on the Cyclus2.

### 2.7. Training Program 

The short-term strength training program, focused on the lower limbs, was based on the latest guidelines of the American College of Sports Medicine [28]. All subjects attended 12 training sessions at the gym, during the 4-week program. Training took place 3 times a week (on Monday, Wednesday, and Friday); each session started at 4 pm, lasted approximately 50 min and included an individual 15 min warm-up: 5 min on a cycle ergometer (XC530 York Fitness, Cardiofit 220 p, Daventry, UK), and 10 min of dynamic stretching; the main part (individual workload); and stretching (5 min of static stretching exercises for the lower limbs). The 1-RM strength test of a leg extension, hamstring curl, and leg press was determined by the use of the protocol from Ammar et al. [29], and it was assessed before, half-way through, and at the end of the program, in order to determine/update the individual load for subjects. This exercise mainly targeted the hamstrings, quadriceps, and gluteus maximus muscles. The leg press exercise was executed with a protocol according to Padulo et al. [30]. Leg extension, hamstring curl, and leg press were performed in the same order, during each training session. All training sessions were performed under the supervision of an experienced trainer, specialized in motor preparation. 

The training exercise load during the first two weeks was 70% of 1-RM. The participants performed 3 sets in the first week and 4 sets in the second one. Each set consisted of 8 repetitions, a 3 min rest between sets in the first week and a 2.5 min break in the second week. The last two weeks of the training protocol consisted of 80% of 1-RM load, 3 sets × 6 repetitions in the third week (3 min rest between sets) and 4 sets × 6 repetition in the fourth week (2.5 min rest between sets). 

### 2.8. Whole-Body Cryostimulation

The CRY group participated in 12 sessions of cold exposure in a cryogenic chamber at the Pomeranian Rheumatology Center in Sopot, Poland. Cryostimulation was conducted 3 times per week (on Tuesday, Thursday, and Saturday) between 8 and 10 a.m., under medical supervision. All sessions lasted 3 min at the temperature of −110 °C. Each entry to the cryogenic chamber was preceded by an adaptation period in the vestibule, at the temperature of −60 °C (for approximately 30 s). Participants wore shorts, socks, and gloves, and hats were covering their auricles [11].

### 2.9. Blood Samplings and Biochemical Assays

Blood samples were taken at rest, 2 h, and 24 h after each EIMD bout (Figure 1). Samples were collected from the antecubital vein into tubes for single use. All samples were immediately placed at 4 °C (after clotting, for serum-separating tubes); they were centrifuged at 2000× *g* for 10 min. Serum aliquots were stored at −80 °C. Serum concentrations of IL-6, IL-15, BDNF, IGF-1, and myostatin were measured using sandwich ELISA kits (R&D Systems, Minneapolis, MN, USA) according to the manufacturer’s protocol (catalog no. HS600B, D1500 DBD00, DG100, and DGDF80, respectively). Serum irisin concentration was determined using a competitive enzyme immunoassay kit from Phoenix Pharmaceuticals, Inc. (Burlingame, CA, USA, catalog no. EK 067-29). To establish muscle damage extent after EIMD bouts, we decided to measure the serum myoglobin level [31], which was determined by ELISA (Wuhan EIAab Science Co. Wuhan, China, catalog no. E048h). 

### 2.10. Statistical Analysis

Statistical analysis was performed using the Statistica 13.1 software (StatSoft, Tulsa, OK, USA). All values are expressed as mean ± standard deviation (SD) and 95% confidence intervals (95% CI). The Shapiro–Wilk test was applied to the data to assess the homogeneity of dispersion from the normal distribution. The Brown–Forsythe test was used to evaluate the homogeneity of variance. Then, separate 2 (group: CRY, CON) × 3 (time: PRE, after 2 h, and 24 h) repeated analyses of variances (ANOVA) were performed. In case of a significant time × group interaction for variables that did violate the normality assumption, ANOVA for repeated measurements and Tukey’s post hoc honest significant difference test for unequal sample sizes were performed to identify significantly different results; for variables that did not violate the normality assumption, the ANOVA Friedman test and the Dunn–Bonferroni post hoc method were applied. Effect sizes (partial eta-squared, $\eta_{p}^{2}$) were additionally calculated with $\eta_{p}^{2}$ ≥ 0.01 indicating small, ≥0.059 indicating medium, and ≥0.138 indicating large effects [32]. Standardized mean differences (SMD) were calculated for pairwise comparison. The magnitude of the SMD was classified according to the following scale: 0–0.19 = negligible effect; 0.20–0.49 = small effect; 0.50–0.79 = moderate effect; and ≥0.80 = large effect. The probability for an effect being practically worthwhile was calculated following the magnitude-based inference approach, using the following scale: 25–75%, possibly; 75–95%, likely; 95–99.5%, very likely; >99%, most likely. Default probabilities for describing the effect as practically beneficial were set at <0.5% (most unlikely) for harm and >25% (possibly) for benefit. 

## 3. Results

### 3.1. Anthropometric Measurement 

No relevant differences between groups, at baseline, in anthropometric features: body weight 73.7 ± 11.7 kg (95% CI: 68.8–78.6); skeletal muscle mass 32.9 ± 7.5 kg (95% CI: 29.8–36.1); or fat mass 15.3 ± 6.9 kg (95% CI: 12.4–18.2) were noted. Those parameters remained unchanged during the entire observation. The only exception was the visceral fat area. This parameter dropped significantly, among all subjects (from 55.2 ± 24.4 cm^2^, 95% CI: 44.9–65.5 to 51.5 ± 22.5 cm^2^, 95% CI: 42.0–61.0).

### 3.2. Physiological Cost of EIMD, Performed before and after the Intervention

**Figure 2:**
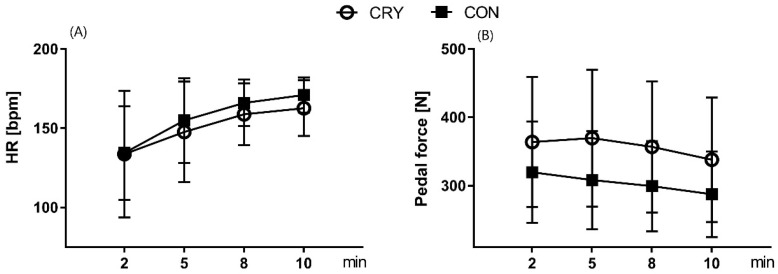
Changes of heart rate (HR) (**A**) and pedal force (**B**) during the second bout of exercise-induced muscle damage. Values between groups were significant different (*p* < 0.05), CRY—cryotherapy group, CON—control group.

All participants performed the first bout of EIMD with an average workload of 331 ± 88 W. At that time, the average pedaling force reached 290 ± 78 N. The main task for subjects was to maintain the exercise intensity. The physiological response, expressed as the HR, gradually increased from 148 ± 37 bpm in the second minute of the exercise, to 162 ± 49 bpm at the end of the bout. After 4 weeks of resistance training and cryostimulation (or passive recovery), the second bout of EIMD was performed with an average workload of 337 ± 85 W. The average HR recorded at the beginning of EIMD was lower (135 ± 36 bpm); yet, changes depended on the group. Differences between the groups’ physiological cost of exercise were especially visible in the eighth minute of EIMD, when the CRY group maintained their HR at 157 ± 12 bpm, and the CON group at 171 ± 9 bpm (Figure 2). The average pedaling force in the CRY group increased to 348 ± 93 N, whereas in the CON group, the average force dropped to 294 ± 93 N (Figure 2).

### 3.3. Exercise-Induced Muscle Damage

**Figure 3:**
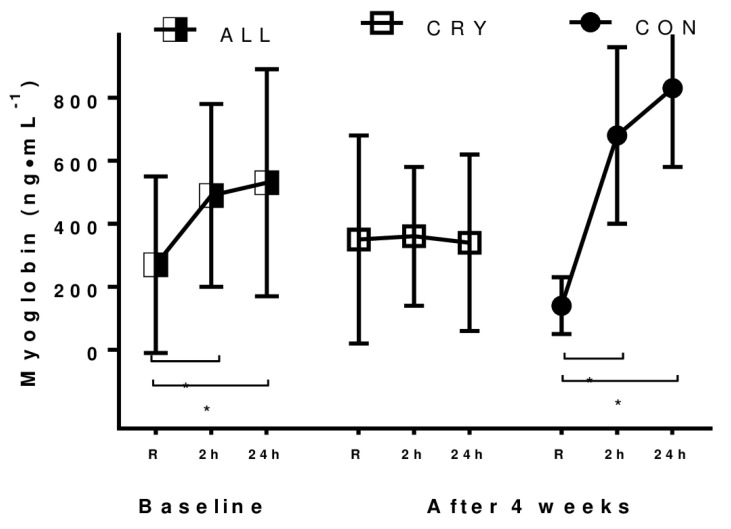
Myoglobin serum concentration at rest and recorded 2 and 24 h post the second bout of exercise-induced muscle damage. Differences between measurements were significant (*p* < 0.05), CRY—cryotherapy group, CON—control group.

The first bout of eccentric cycling induced muscle damage in all participants. This observation was based on a high level of pain sensation and muscle stiffness as well as on serum myoglobin levels, which were noticed 2 h after the exercise and persisted for 24 h after finishing the bout. Subjects in both groups assessed their pain using the VAS scale as 3 ± 2, 2 h directly after the exercise and 6 ± 2 24 h after finishing the bout. The recorded pain sensation did not correspond to an average increase of the serum myoglobin level. The first bout of EIMD induced a significant rise of myoglobin among all participants (baseline vs. 2 h post *p* = 0.05, baseline vs. 24 h post *p* = 0.00) (Figure 3). After 4 weeks of training in the CON group, a significant increase of myoglobin was observed (baseline vs. 2 h post *p* = 0.02, baseline vs. 24 h post *p* = 0.01), whereas the CRY group was characterized by low myoglobin, which remained unchanged (Figure 3). The second bout of EIMD did not trigger a similar perception of pain. Compared to the first bout, both groups experienced a lower perceived pain 24 h post-exercise. However, on the VAS scale, perceived pain was still higher in the CON group (5 ± 2) compared to that in the CRY group (2 ± 3). Differences were significant (*p* = 0.05).

### 3.4. Exerkine Response to Training and Treatment

The first bout of eccentric exercise did not cause significant changes in the concentration of assayed myokines and growth factors. Due to the fact that cryotherapy sessions were introduced just one day after performing the first EIMD, those values are not presented. 

**Figure 4:**
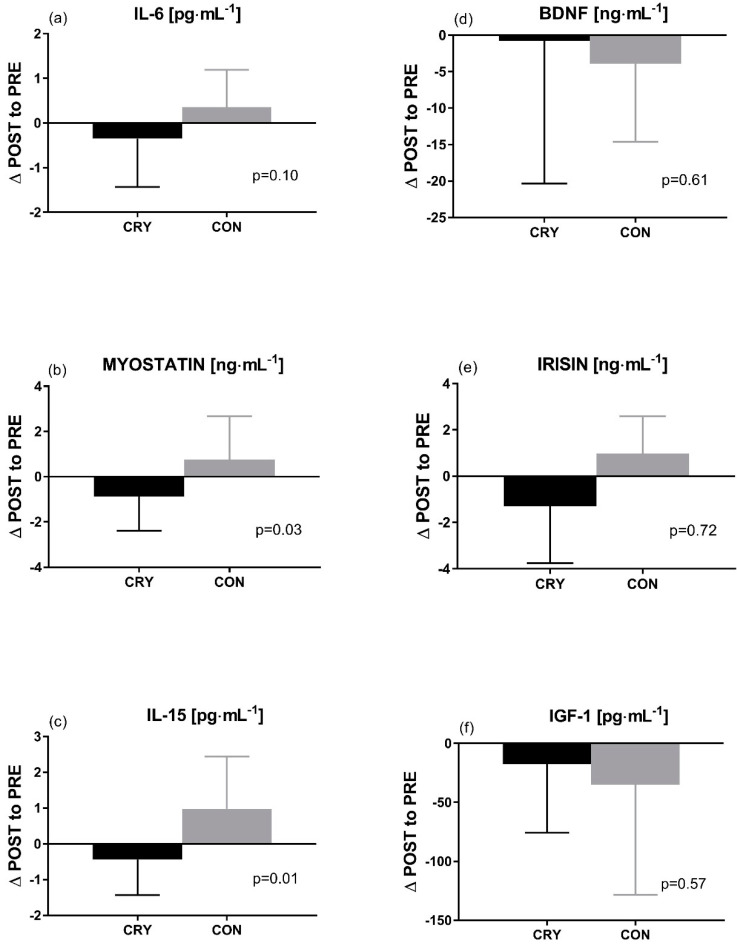
Delta changes (∆ POST to PRE) in serum myokine and growth factor concentrations at rest: before and after 4 weeks of resistance training combined with whole-body cryostimulation or passive rest; (**a**) interleukin 6 (IL-6), (**b**) myostatin, (**c**) interleukin 15 (IL-15), (**d**) brain-derived neurotrophic factor (BDNF), (**e**) irisin, (**f**) insulin-like growth factor (IGF-1); POST—post-intervention values, PRE—pre-intervention values, CRY—cryotherapy group, CON—control group; *p* < 0.05. The probability of an effect being practically worthwhile was calculated according to the magnitude-based inference method.

The applied training response varied between the CRY and CON groups expressed in resting values of measured indicators (Figure 4). There was a significant and large interaction between group × time after 4 weeks of training for myostatin (*p* = 0.03, $\eta_{p}^{2}=0.20$) and IL-15 (*p* = 0.01, $\eta_{p}^{2}=0.27)$, (Figure 4b,c). A similar tendency was registered for irisin (Figure 4e), but the effect was not significant (*p* = 0.72, $\eta_{p}^{2}=0.00$). The IGF-1 level decreased in both groups; however, the magnitude of change was more pronounced in the CON group (*p* = 0.57, $\eta_{p}^{2}=0.01$) (Figure 4f). Levels of IL-6 and BDNF remained unchanged across the observation (*p* = 0.10, $\eta_{p}^{2}=0.11$; *p* = 0.61, $\eta_{p}^{2}=0.01$), respectively) (Figure 4a,d). 

The correlations among the changes in muscle function and IGF-1 were investigated. The growth factor IGF-1 positively correlated with the peak torque maximal isokinetic 90°·s^−1^ (Nm) (r = 0.62, *p* = 0.04), and the average power isokinetic 90°·s^−1^ (Nm) (r = 0.54, *p* = 0.04). In the CON group, the correlations had an opposite tendency and were not significant (r = −0.32, *p* = 0.37; r = −0.28, *p* = 0.37, respectively). 

The second bout of EIMD triggered different responses in exerkines and growth factors, depending on the recovery strategy applied. This effort induced a significant increase of myostatin concentration in the CON group (Table 1), while in the CRY group, myostatin was unaffected over the entire post-exercise observation (the difference between groups was significant, *p* = 0.27, $\eta_{p}^{2}=0.05)$. The second bout of EIMD triggered a significant drop of BDNF in all participants (noticed 2 h as well 24 h post exercise), still the magnitude of this change was greater in the CON group in comparison to that in CRY (*p* = 0.00, $\eta_{p}^{2}=0.16)$. A similar trend was noted in IGF-1 concentration, but in this case, the magnitude of change was not that noticeable (*p* = 0.86, $\eta_{p}^{2}=0.01)$. A different IL-15 response was noted among two groups. In the CRY group, IL-15 concentration increased only 2 h after the last eccentric bout, whereas its concentration decreased in the CON group and remained low, until 24 h after EIMD (*p* = 0.21, $\eta_{p}^{2}=0.07)$. Changes in the irisin levels were not significant (*p* = 0.57, $\eta_{p}^{2}=0.02)\;$(Table 1). 

### 3.5. Changes in Muscle Strength after the Intervention

The applied procedure of resistance training resulted the improvement of workload at 1-RM in all participants (20% for hamstring-curl, 19% for leg-extension, and 11% for leg press).

The effect of the training program associated with different recovery strategies on strength in isometric and isokinetic conditions is shown in Table 2. Training combined with CRY induced significant increase of maximal average power in the knee isokinetic extension strength test, SMD: 0.42 (left leg), 0.32 (right leg), while in the control group the level of strength has remained stable, SMD: 0.11 (left leg), 0.03 (right leg). Similar, but not significant, changes have been observed in maximal peak torque in the knee isokinetic extension strength test, SMD: 0.18 (left leg), 0.15 (right leg), where at the same time, in the control group, a decrease has been noted, SMD: 0.06 (left leg), 0.22 (right leg). An adverse tendency was registered in isometric strength.

## 4. Discussion

To the best of our knowledge, this is the first study to evaluate the impact of short-term resistance training combined with a whole-body cryostimulation protocol, on muscle strength and circulating biomarkers related to muscle damage and recovery. Data recorded after the 4-week intervention demonstrated improvement in average power and isokinetic extension muscle strength. These changes were accompanied by shifts in exerkines concentrations: a drop in myostatin and a growth in IL-15 concentration in the participants subject to both resistance training and cold treatment. Morton et al. found recently that an acute response of circulating levels of growth factors did not correlate with functional changes in muscles (i.e., strength) following resistance training [33]. This suggests other factors must have contributed to the training-induced adaptations. The current study, for the first time, revealed that the effectiveness of whole-body cryostimulation is manifested in a lower circulating level of myostatin, a negative regulator of muscle hypertrophy [34].

Burd et al. have reported [35] that a workload of as little as 30% and up to 90% of 1-RM played a minimal role in stimulating muscle protein synthesis and altered greater motor unit recruitment. Conversely, low temperatures are known to result in lower nerve conduction velocities [36]. In our study, isokinetic muscle strength increased. Since no changes in muscle mass were recorded, this increase can be connected with better motor unit recruitment.

At the same time, positive changes in muscle strength were accompanied by a drop of myostatin. The effects of this protein are mediated by inhibition of the Akt kinase activity, which can lead to the FOXO3a transcription factor being activated, which in turn induces expression of atrogin-1 encoding for a protein strongly linked to muscle atrophy [37]. A decline in myostatin was recorded after 4 weeks of strength training and cold treatment as well as within 2 and 24 h after the second bout of EIMD. Literature has characterized the influence of exercise on myostatin concentration as ambiguous. Kazemi et al. reported that a single session of high-volume circuit resistance training resulted in a significant decrease in plasma myostatin 24 h after exercise [38]. Contrarily, according to Willoughby et al., a single session of eccentric muscle actions caused an increase in serum myostatin concentration with peak values recorded 24 h post exercise [39]. Such divergence of effects was also reported in response to regular resistance training. A total of 4 weeks of whole-body resistance training caused a significant decline in myostatin levels, independently of the administered creatine supplementation [40]. At the same time, Willoughby documented increased myostatin concentrations after 12 weeks of lower-body resistance training [41]. A recently published paper indicated that a 3 h exposure to 7 °C temperature did not affect myostatin gene expression [42]. In a separate study, the aforementioned protocol of whole-body cryostimulation, combined with specific volleyball training, had no impact on myostatin concentration [20]. Therefore, we concluded that the specific response to resistance training and cold treatment depended on the presence of a specific exercise stimulus. Alternatively, a drop in myostatin concentration could have been connected with the discovery by Kong et al., who showed that skeletal muscle and brown adipose tissue (BAT) are functionally interlinked [43]. They described an intriguing role of the transcription factor interferon regulatory factor 4 (IRF4) in BAT, whereby it had mediated BAT-muscle crosstalk through myostatin. Thermoneutrality or loss of IRF4 were found to have resulted in elevated serum myostatin levels and decreased exercise capacity [43]. Although, we did not measure BAT, exposure coldness is known to considerably affect its metabolic activity [44].

Previously published data revealed that myostatin can also modulate metabolic homeostasis by regulating adipose tissue function [45]. In his review, Huh argued that myostatin modified irisin concentration, acting through adipocyte browning and subsequent induction of energy expenditure [24]. In our investigation, irisin was not affected in response to cryostimulation, contrarily to previous observations [46]. These results may be linked to the lower levels of adipose tissue exhibited by the subjects of the current study, or even the frequency of whole-body cryostimulation (3 times per week) in combination with the training program.

In the present study, a decrease in myostatin levels in the CRY group was accompanied by a decline in IGF-1. In a previous study, an increased level of IGF-1 was recorded after a single session of acute resistance exercise as well as in response to 8 weeks of resistance training [47]. According to recent findings, myostatin and IGF-1 regulate skeletal muscle size and myofiber type expression through different mechanisms [48]. In response to the applied intervention, these two hormones followed opposite trends of change, ascending and descending, respectively, depending on the recovery strategy applied. In myostatin null mice, a decreased *Igf-1* mRNA expression in skeletal muscles was observed, with the difference being greater in younger than in older mice [49]. Based on these findings, it was suggested that myostatin regulated hyperplasia, while IGF-1 regulated hypertrophy of myofibers, with the two processes being temporarily separated [49]. It is worth considering that this decrease of IGF-1 occurred due to an increase of IGF-binding proteins (IGFBPs). The same researchers demonstrated that myostatin knock-out caused expression of *Igfbp5* mRNA to grow [49] despite previous studies reporting contradictory results [50]. In the context of whole-body cryostimulation, the treatment is associated with an increase of blood catecholamine levels [51], which in turn is associated with an increase of IGFBP-1 in humans [50]. The lack of IGFBP-1 in blood analysis can, thus, be considered as a limitation of this study and should be included in future investigations.

Although muscle mass was not affected in response to the applied training program, significant changes were noted in IL-15 concentration among the CRY subjects. IL-15 was shown to have an impact on muscle metabolism and hypertrophy [24]. Perez-Lopez et al. demonstrated that a single session of 4 sets of leg press and leg extension at 75% 1-RM stimulated the IL-15/IL-15Rα signaling pathway together with an elevated serum concentration of IL-15. They also observed that the activation of this signaling pathway supported myofibrillar protein synthesis [52]. Nevertheless, this was only the effect of a single unit of exercise without any recovery-supporting method. In a previous investigation, a specific volleyball training program combined with cryostimulation did not significantly affect the concentration of IL-15 [20]. In our study, 12 units of regular resistance training with a workload comparable to the Perez-Lopez study, but combined with cryostimulation, caused a significant increase of IL-15 resting concentration. The obtained results may indicate that cold treatment did not attenuate synthesis of IL-15.

We also measured BDNF concentration—an exercise-induced growth factor with a major impact on the nervous system including a protective role for the central and peripheral neurons [53]. Some studies, however, highlighted the metabolic role of BDNF in regulating energy homeostasis [54], which may have a positive effect on skeletal muscle adaptation to resistance training. Despite this, resistance training was reported not to have affected the basal circulating level of BDNF in comparison to aerobic training [55]. A recently published investigation confirmed this observation [56]. Until now, only one study observed an increase of BDNF concentration in response to volleyball training combined with whole-body cryostimulation [20]. Results obtained in this study indicate that resistance training combined with whole-body cryostimulation did not affect the BDNF level, which can suggest that neither resistance training alone nor in combination with cold treatment affects BDNF concentration.

Previous studies showed that submaximal-intensity eccentric training, performed for 4–8 weeks in between acute EIMD bouts, contributed to an ameliorated systemic response to the second EIMD bout in young active participants [57,58]. Our results show that resistance training may have had a similar effect. The increased muscle strength would have also contributed to a reduced physiological cost of the second bout of EIMD. The CRY group exhibited lower values of myoglobin concentration than those recorded in the CON group, which agrees with previously published studies showing that whole-body cryostimulation may reduce circulating enzymes related to muscle damage, such as creatine kinase and lactate dehydrogenase [59,60]. Our study examined 12 sessions of high-intensity resistance training in combination with either whole-body cryostimulation or passive recovery. According to a review by Damas, the applied number of training units could have been insufficient to induce muscle hypertrophy [6]. However, our data suggest that cryostimulation was effective in lowering the concentration of myostatin in the early phase of resistance training and, hence, allow to conclude that the number of training units applied may have been sufficient to induce muscle remodeling and to trigger the process of muscle adaptation to training.

In the context of the reported results and findings, limitations of the study should be discussed. Firstly, the analyses’ week’s training program only showed the first step of adaptation to a training process; hence, the impact of long-term resistance training combined with whole-body cryostimulation should still be investigated further. Secondly, the applied resistance training program started shortly after the first bout of EIMD. Thus, measuring the size of muscle damage may not have been justified even though we were able to measure muscle damage at 48 and 72 h after the first bout.

## 5. Conclusions

The whole-body cryostimulation combined with resistance training can positively modify concentrations of growth factors among untrained subjects by reducing myostatin concentration. This type of cold treatment, applied a day after a high-intensity resistance training session, did not inhibit muscle adaptation to resistance training among beginners. Thus, the time difference in treatment application should be taken into consideration in future investigations to formulate precise conclusions regarding combining cold therapy with resistance training.

## Figures and Tables

**Table 1:** Exerkines concentration before and after the second bout of exercise-induced muscle damage performed after 4 weeks of resistance training combined with cryostimulation or passive rest.

Exerkines	Group	PRE Second EIMD		POST After 2 h		SMD	MBI	POST After 24 h		SMD	MBI	ANOVA *p*$\;(\eta_{p}^{2})$
		$\bar{X}$ ± SD	95% CI	$\bar{X}\;$ ± SD	95% CI	Cohen’s d	Practically Worthwhile Effect	$\bar{X}\;$ ± SD	95% CI	Cohen’s d	Practically Worthwhile Effect	Group × Time Interaction
**IL-6**	CON	1.3 ± 0.6	0.9–1.7	1.1 ± 0.5	0.7–1.5	0.36	possible	0.9 ± 0.4	0.6–1.2	**0.80**	unclear	0.42 (0.04)
(**pg∙mL^−1^**) **^†^**	CRY	1.4 ± 1.1	0.8–2.0	1.4 ± 1.1	0.8–2.0	0.00		0.9 ± 0.7	0.5–1.3	**0.56**		
**Myostatin**	CON	5.1 ± 1.8	3.8–6.4	6.2 ± 1.2	5.3–7.1 *	**0.73**	likely	5.7 ± 1.4	5.0–6.4	0.38	likely	0.27 (0.05)
(**ng∙mL^−1^**) **^†^**	CRY	3.8 ± 1.8	2.8–4.8	3.8 ± 2.2	2.6–5.0	0.00		3.9 ± 1.1	3.3–4.5	0.07		
**IGF-1**	CON	173.6 ± 69.6	123.8	167.8 ± 68.7	118.7–216.9	0.08	unclear	162.2 ± 77.2	107.0–217.4	0.16	unclear	0.86 (0.01)
(**pg∙mL^1^**) **^†^**	CRY	122.2 ± 50.7	94.1–150.3	118.8 ± 42.9	95.0–142.6	0.07		115.8 ± 44.4	91.2–140.4	0.13		
**IL-15**	CON	1.7 ± 1.4	0.7–2.7	1.1 ± 0.9	0.5–1.7	**0.52**	possible	0.9 ± 0.8	0.3–1.5	**0.73**	unclear	0.21 (0.07)
(**pg∙mL^−1^**) **^†^**	CRY	1.2 ± 0.6	0.9–1.5	1.6 ± 1.7	0.7–2.5	0.35		1 ± 0.6	0.7–1.3	0.33		
**BDNF**	CON	42.6 ± 14.6	32.2–53.0	25.4 ± 9.6 *	18.5–32.3	**1.42**	unclear	37.3 ± 6.1 *	32.9–41.7	**0.51**	likely	**0.00 (0.16)**
(**ng∙mL^−1^**)	CRY	40.4 ± 9.6	35.1–45.7	32.8 ± 13.4 *	25.4–40.2	**0.66**		32.1 ± 12.9 *	25.0–39.2	**0.74**		
**Irisin**	CON	3.8 ± 4.5	0.6–7.0	3.6 ± 4	0.7–6.5	0.05	possible	3.2 ± 3.2	0.9–5.5	0.16	possible	0.57 (0.02)
(**ng∙mL^−1^**) **^†^**	CRY	1.8 ± 1.7	0.9–2.7	1.9 ± 1.7	0.9–2.8	0.06		1.8 ± 1.7	0.9–2.7	0.00		

Note: Values are presented as mean ± SD; ^†^ nonparametric analysis; 95% CI—confidential interval, CON—control group, CRY—cryotherapy group; EIMD—exercise-induced muscle damage; * significant differences from pre-second EIMD level, *p* < 0.05. Moderate to large standardized mean differences (SMD) have been highlighted in bold. Eta-squared effects (η^2^_p_) for interaction are presented in brackets. The probability of an effect being practically worthwhile was calculated according to the magnitude-based inference (MBI) method. Unclear effect means that more data are needed to assess the intervention effect.

**Table 2:** The effect of 4 weeks of resistance training combined with different recovery procedures on extension maximal isometric and isokinetic measurements.

		Pre Intervention Level		Post Intervention Level		SMD	$\mathrm{ANOVA}\;p\;(\eta_{p}^{2})$	MBI
		$\bar{X}$ ± SD	95% CI	$\bar{X}$ ± SD	95% CI	Cohen’s d	Group × Time Interaction	Practically Worthwhile Effect
**PT max isometric [Nm] extension**								
Left leg	CON	207 ± 56	167–247	208 ± 59	166–250	0.02	0.08 (0.13)	possible
	CRY	222 ± 71	183–261	208 ± 63 *	173–243	0.21		
Right leg	CON	201 ± 60	158–244	203 ± 63	158–248	0.03	0.69 (0.01)	unclear
	CRY	217 ± 58	185–249	215 ± 59	182–248	0.03		
**PT max isokinetic 90° s^−1^ [Nm] extension**								
Left leg ^†^	CON	144 ± 32	121–167	142 ± 31	120–164	0.06	0.17 (0.08)	likely
	CRY	167 ± 46	142–192	175 ± 41	152–198	0.18		
Right leg	CON	156 ± 39	128–184	148 ± 33	124–172	0.22	0.05 (0.16)	likely
	CRY	166 ± 46	141–191	173 ± 45	148–198	0.15		
**AP max isokinetic 90° s^−1^ [W] extension**								
Left leg ^†^	CON	129 ± 27	110–148	132 ± 27	113–151	0.11	0.13 (0.10)	very likely
	CRY	153 ± 53	124–182	173 ± 43 *	149–197	0.42		
Right leg	CON	141 ± 31	119–163	142 ± 29	121–163	0.03	0.10 (0.11)	likely
	CRY	157 ± 51	129–185	173 ± 50 *	145–201	0.32		

Note: Values are presented as mean ± SD; ^†^ nonparametric analysis; 95% CI—confidential interval, PT max—peak torque maximal, AP max—average power maximal, CON—control group, CRY—cryotherapy group; * significant differences from pre-intervention level, *p* < 0.05. Moderate-to-large standardized mean differences (SMD) have been highlighted in bold. Eta-squared effects (η^2^_p_) for interaction are presented in brackets. The probability of an effect being practically worthwhile was calculated according to the magnitude-based inference (MBI) method. Unclear effect means that more data are needed to assess the intervention effect.
